# Two phase I studies of BI 836880, a vascular endothelial growth factor/angiopoietin-2 inhibitor, administered once every 3 weeks or once weekly in patients with advanced solid tumors

**DOI:** 10.1016/j.esmoop.2022.100576

**Published:** 2022-09-13

**Authors:** C. Le Tourneau, H. Becker, R. Claus, E. Elez, F. Ricci, R. Fritsch, Y. Silber, A. Hennequin, J. Tabernero, G. Jayadeva, D. Luedtke, M. He, N. Isambert

**Affiliations:** 1 Department of Drug Development and Innovation (D3i), Institut Curie, INSERM U900 Research Unit, Paris-Saclay University, Paris & Saint-Cloud, France; 2 Department of Medicine I (Hematology, Oncology and Stem Cell Transplantation), Medical Center – University of Freiburg, Faculty of Medicine, University of Freiburg, Freiburg, Germany; 3 Medical Faculty Augsburg University, Augsburg, Germany; 4 Vall d’Hebron Barcelona Hospital Campus and Vall d’Hebron Institute of Oncology (VHIO), Barcelona, Spain; 5 Autonomous University of Barcelona, Barcelona, Spain; 6 Centre Georges-François Leclerc, Dijon, France; 7 UVic-UCC, IOB-Quiron, Barcelona, Spain; 8 Boehringer Ingelheim International GmbH, Ingelheim am Rhein; 9 Boehringer Ingelheim Pharma GmbH and Co KG, Biberach, Germany; 10 Boehringer Ingelheim Pharmaceuticals Inc., Ridgefield, USA

**Keywords:** Vascular endothelial growth factor, angiopoietin-2, advanced solid tumors, phase I, nanobody

## Abstract

**Background:**

BI 836880 is a humanized bispecific nanobody® that inhibits vascular endothelial growth factor and angiopoietin-2. Here, we report results from two phase I, nonrandomized, dose-escalation studies (NCT02674152 and NCT02689505; funded by Boehringer Ingelheim) evaluating BI 836880 in patients with confirmed locally advanced or metastatic solid tumors, refractory to standard therapy, or for which standard therapy was ineffective.

**Patients and Methods:**

Patients aged ≥18 years, with an Eastern Cooperative Oncology Group performance status of 0-2 and adequate organ function received escalating intravenous doses of BI 836880 once every 3 weeks (Q3W; Study 1336.1) or once weekly (QW; Study 1336.6). Primary objectives were maximum tolerated dose (MTD) and recommended phase II dose of BI 836880, based on dose-limiting toxicities (DLTs) during the first cycle.

**Results:**

Patients received one of five dosages of 40-1000 mg Q3W (29 patients) or 40-240 mg QW (24 patients). One DLT occurred with Q3W treatment [Grade (G) 3 pulmonary embolism (1000 mg)]. Five DLTs occurred in four patients treated QW [G2 proteinuria (120 mg); G3 hypertension (180 mg); G3 proteinuria and G3 hypertension (240 mg); and G4 respiratory distress (240 mg)]. All patients experienced adverse events, most commonly hypertension with Q3W treatment (89.7%; G3 41.4%), and asthenia with QW treatment (62.5%). Two patients treated Q3W (both 1000 mg) and three patients treated QW (120 mg, 2 patients; 180 mg, 1 patient) experienced partial response.

**Conclusions:**

The MTD of BI 836880 was 720 mg Q3W and 180 mg QW. BI 836880 was generally manageable and demonstrated preliminary efficacy.

**Clinical trial registration:**

ClinicalTrials.govNCT02674152; https://clinicaltrials.gov/ct2/show/NCT02674152 and NCT02689505; https://clinicaltrials.gov/ct2/show/NCT02689505

## Highlights

• BI 836880 is a humanized bispecific nanobody® that inhibits vascular endothelial growth factor and angiopoietin-2.
• Here, we evaluate BI 836880 in 3 + 3 phase I studies in patients with confirmed locally advanced or metastatic solid tumors.
• The maximum tolerated dose of BI 836880 was reached (720 mg once every 3 weeks and 180 mg once weekly).
• BI 836880 was generally manageable and preliminary antitumor activity was observed.
• Ongoing studies are evaluating BI 836880 and other agents combined with programmed cell death protein-1 inhibitor ezabenlimab.

## Introduction

Angiogenesis, the process by which new blood vessels are formed from pre-existing vasculature, is one of the hallmarks of cancer.^1^^,^^2^ Vascular endothelial growth factor (VEGF) is a proangiogenic growth factor that is often overexpressed in cancer.^3^ Targeting VEGF is an established anticancer therapeutic approach. The anti-VEGF-A monoclonal antibody, bevacizumab, was first approved over 15 years ago, and is now used in the treatment of a range of solid tumor types.^4^ Other agents targeting angiogenesis, such as the antivascular endothelial growth factor receptor-2 (VEGFR-2) antibody ramucirumab, the recombinant fusion protein aflibercept, and VEGF receptor tyrosine kinase inhibitors, are also approved for the treatment of a variety of advanced cancers.^5^^,^^6^

While the activity of VEGF and the therapeutic value of VEGF-signaling inhibition have been established, the contribution of other proangiogenic pathways and the clinical impact of selective inhibition of those other targets are less well described. One such target is the angiopoietin/Tie signaling pathway, which plays a role in vascular stability and acts in a complementary manner to VEGF.^7^ Angiopoietin-2 (Ang-2) inhibits angiopoietin-1-induced Tie-2 signaling and promotes vessel remodeling, which is necessary for VEGF-induced angiogenesis.^8^^,^^9^ There is also crosstalk between the two pathways as Ang-2 enhances VEGF signaling, and VEGF upregulates Ang-2 expression.^9^^,^^10^

VEGF and Ang-2 also have distinct immunosuppressive effects in the tumor microenvironment.^11^ Thus, inhibiting VEGF and Ang-2 may also enhance the tumor microenvironment to support T cell function, providing a rationale for combining with agents that target the programmed death protein-1 (PD-1) pathway.

Clinical studies have demonstrated that patients with tumors harboring high expression levels of both VEGF and Ang-2 have poorer prognoses than patients with high expression of only one of these proteins.^12^^,^^13^ Therefore, it has been suggested that dual inhibition of VEGF and Ang-2 may improve clinical activity in comparison to the inhibition of either pathway alone. This is supported by data from preclinical models.^7^^,^^14^

BI 836880 is a humanized bispecific nanobody® comprising two single variable domains that bind to all splice variants of VEGF (VEGF-165, VEGF-121, and VEGF-189) and Ang-2, inhibiting binding of VEGFR-2 and Tie-2. It also contains an additional albumin module that extends half-life *in vivo.*^9^ BI 836880 potently and selectively neutralizes VEGF and Ang-2 and showed superior antitumor activity to VEGF or Ang-2 inhibition alone in preclinical models of cancer.^9^

Here we report results from two phase I studies of BI 836880 in patients with advanced or metastatic solid tumors. Study 1336.1 assessed BI 836880 given once every 3 weeks (Q3W), and Study 1336.6 assessed BI 836880 given once weekly (QW). The primary objective of each study was to define the maximum tolerated dose (MTD) and recommended phase II dose (RP2D) of BI 836880 in patients with advanced or metastatic solid tumors.

## Methods

### Study design and patients

Both studies were phase I, noncontrolled, nonrandomized, open-label, dose-escalation studies. Study 1336.1 (NCT02674152) was conducted at three centers in France and Germany (January 2016 to September 2018) and Study 1336.6 (NCT02689505) was conducted at two centers in France and Spain (May 2016 to July 2019). Both studies were sponsored by Boehringer Ingelheim.

The studies were conducted in accordance with the Declaration of Helsinki and Good Clinical Practice guidelines as defined by the International Conference on Harmonization; all patients provided written informed consent for study participation.

Eligible patients in both studies were aged ≥18 years, with confirmed locally advanced or metastatic solid tumors that were either refractory after standard therapy or for which standard therapy was not reliably effective. Patients were also required to have an Eastern Cooperative Oncology Group performance status of 0-2; adequate organ function; a life expectancy of ≥3 months; and had recovered from all reversible adverse events (AEs) of previous anticancer therapies to baseline (except alopecia, sensory peripheral neuropathy, and those not considered clinically significant) or Common Terminology Criteria for Adverse Events (CTCAE) Grade 1.

Exclusion criteria included: known hypersensitivity to the study drug; prior treatment with any systemic anticancer therapy within 28 days; serious concomitant disease; surgery/major injury within 4 weeks; QT prolongation and/or long QT syndrome; or symptomatic brain metastases. Considering the mechanism of action, patients with significant cardiovascular or cerebrovascular diseases (i.e. uncontrolled hypertension, unstable angina, history of infarction within the past 6 months, congestive heart failure classification of at least New York Heart Association class II); a severe hemorrhagic or thromboembolic event in the past 12 months; and patients who required full-dose anticoagulation were also excluded.

### Treatment

In both studies, patients received BI 836880 via intravenous infusion at a starting dose of 40 mg (Q3W in 1336.1 and QW in 1336.6). Dose escalation was guided by a Bayesian logistic regression model (BLRM) utilizing escalation with overdose control (EWOC).

The BLRM estimated the MTD by updating estimates of the probability of observing a dose-limiting toxicity (DLT) in the MTD evaluation period for each dose level as data accumulated. The dose of BI 836880 was escalated in cohorts at predefined provisional dose levels based on a maximum escalation of 200% for doses ≤120 mg Q3W and ≤40 mg QW and 100% for doses >120 mg Q3W or QW. Successive cohorts of patients received increasing doses of BI 836880 until the MTD was reached.

Once the MTD had been reached, this dose level was expanded to further examine safety and further endpoints at this dose. Treatment was continued until disease progression or unacceptable toxicity.

### Endpoints and assessments

The primary endpoint in both studies was the MTD of BI 836880 and the number of patients experiencing DLTs during the MTD evaluation period (first 3-week cycle). Secondary endpoints included drug-related AEs (DRAEs) leading to dose reduction or discontinuation; exposure measures [area under the plasma concentration–time curve from time zero to the last quantifiable concentration (AUC_0–tz_)]; and disposition kinetic measures [elimination half-life (*t*_1/2_)] after the first dose. Further endpoints included best overall response.

AEs were graded according to CTCAE version 4.03. DLTs are defined in Supplemental Materials S1, available at https://doi.org/10.1016/j.esmoop.2022.100576. MTD was defined as the highest dose with <25% risk of the true DLT rate being above 0.33 during the MTD evaluation period, and could be considered reached if there was a sufficiently large probability that the true DLT rate was in the target interval of 0.16-0.33. RP2D was selected based on an analysis of overall safety, and pharmacokinetic (PK) and pharmacodynamic (PD) data.

Serial blood samples were collected to determine plasma levels of BI 836880, free VEGF-A and free/total levels of Ang-2 and antidrug antibodies (ADAs). BI 836880 plasma concentrations were quantified using a validated bioanalytical assay. Plasma biomarker analyses of free/total VEGF-A and Ang-2 were performed using immunoassays and the presence of ADAs to BI 836880 was assessed via a tiered approach using a validated electrochemiluminescence assay (MSD QuickPlex SQ 120 Reader; Meso Scale Discovery® Model SQ 120).

Tumor response was assessed every 6 weeks and evaluated by the investigator, per Response Evaluation Criteria in Solid Tumors (RECIST version.1.1) and was defined as the best response at any time from the date of the first administration of the study drug until progression.

Patients were followed up until disease progression, start of a new anticancer therapy, death, end of the study, or lost to follow-up.

### Statistical analysis

Dose escalation was guided by a two-parameter BLRM with overdose control. The estimated probability of a DLT at each dose level from the model was summarized using the following intervals: under toxicity [0.00, 0.16]; targeted toxicity [0.16, 0.33]; and over toxicity [0.33, 1.00]. The BLRM-recommended dose for the next cohort was the level with the highest posterior probability of the DLT rate falling in the target interval [0.16, 0.33] among the doses fulfilling the EWOC criterion. According to the EWOC criterion, it should be unlikely (<25% posterior probability) that the DLT rate at that dose is ≥0.33.

Analyses of AEs were descriptive and the relationship between the study drug and the AE was assessed by the investigator. The best overall response and ADA levels were analyzed descriptively.

PK parameters were calculated using noncompartmental analysis via Phoenix WinNonlin. A population PK and integrated PD model was developed based on data from patients treated Q3W to support dose selection for future studies. Population PK and PK/PD analyses for repeated-measures endpoints were conducted via nonlinear mixed effects modeling with a qualified installation of the nonlinear mixed effects modeling (NONMEM) software.

## Results

### Patients and treatment

Overall, 29 patients received BI 836880 Q3W, and 24 patients received BI 836880 QW. At the time of data cut-off, 27 patients had discontinued Q3W treatment [mainly due to progressive disease (*n* = 19)] and 2 patients remained on treatment. All patients had discontinued the QW treatment schedule [mainly due to progressive disease (*n* = 17)]. Across both studies, no patients withdrew consent (Supplementary Figure S1, available at https://doi.org/10.1016/j.esmoop.2022.100576).

Baseline characteristics for patients treated with the Q3W and QW schedules are presented in Table 1. In patients receiving treatment Q3W, the median (range) age was 57.0 (28-79) years and the majority of patients were female (62.1%). In patients receiving the QW treatment schedule the median (range) age was 58.5 (31-74) years and the majority of patients were also female (70.8%).

**Table 1:** Baseline characteristics for both studies (treated set)

	Q3W (*n* = 29)	QW (*n* = 24)
Median age at baseline, years (range)	57.0 (28-79)	58.5 (31-74)
Gender, *n* (%)		
Male	11 (37.9)	7 (29.2)
Female	18 (62.1)	17 (70.8)
Race, *n* (%)		
White	9 (31.0)	0 (0)
Hispanic/Latino	0 (0)	14 (58.3)
Missing^a^	20 (69.0)	10 (41.7)
Baseline ECOG performance status, *n* (%)		
0	11 (37.9)	17 (70.8)
1	18 (62.1)	7 (29.2)
2	0 (0)	0 (0)
Median time from first diagnosis, years (range)	3.5 (0.3-22.6)	3.8 (0.5-12.6)
Primary cancer diagnosis, *n* (%)		
Colon/rectum	5 (17.2)	10 (41.7)
Pancreas	6 (20.7)	3 (12.5)
Breast	4 (13.8)	1 (4.2)
Esophagus	3 (10.3)	1 (4.2)
Ovary	1 (3.4)	3 (12.5)
Cavum	2 (6.9)	0 (0)
Choroid melanoma	2 (6.9)	0 (0)
Thymus	2 (6.9)	0 (0)
Other	4 (13.8)^b^	6 (25.0)^c^
Prior anticancer therapy, *n* (%)		
Chemotherapy	29 (100.0)	24 (100.0)
Immunotherapy	4 (13.8)	4 (16.7)
Radiotherapy	18 (62.1)	8 (33.3)
Hormone therapy	5 (17.2)	2 (8.3)
Other	10 (34.5)	1 (4.2)
Number of prior systemic therapy regimens, median (range)	2 (1.0-4.0)^d^	1.5 (1.0-3.0)^e^
Prior antiangiogenic treatment, *n* (%)	14 (48.3)	14 (58.3)
Baseline conditions, *n* (%)		
Hypertension	16 (55.2)	8 (33.3)
Anemia	9 (31.0)	0 (0)

ECOG, Eastern Cooperative Oncology Group; Q3W; once every 3 weeks; QW, once weekly.

a Race and ethnicity data were not recorded for patients treated in France, as it was prohibited by local law.

b This category includes one patient each with the primary site of left iliac fossa (neuroectodermal tumor), proximal jejunum, uterus, and unknown origin.

c This category includes one patient each with the primary site of cervix, endometrium, gastric, kidney, peritoneum, and trachea.

d A total of 8 patients had received one prior regimen, 11 patients had received two prior regimens, 4 patients had received three prior regimens, and 6 patients had received four prior regimens.

e A total of 12 patients received one prior regimen, 9 patients had received two prior regimens, and 3 patients had received three prior regimens.

Median duration of treatment was 22.0 days for the Q3W schedule (range 1-786) and 66.5 days for the QW schedule (range 7-524). Mean duration of treatment was 105.5 days for Q3W treatment and 112.4 days for QW treatment.

Patients received BI 836880 at five dose levels from 40 to 1000 mg Q3W (40 mg, *n* = 3; 120 mg, *n* = 2; 360 mg, *n* = 2; 720 mg, *n* = 17; 1000 mg, *n* = 5), and at five dose levels from 40 to 240 mg QW (40 mg, *n* = 2; 120 mg, *n* = 5; 150 mg, *n* = 3; 180 mg, *n* = 11; 240 mg, *n* = 3).

### Determination of the MTD

In patients treated Q3W, one DLT occurred during the MTD evaluation period (Grade 3 pulmonary embolism that led to discontinuation in a patient with pancreatic adenocarcinoma in the 1000 mg group). Although the BLRM allowed expanding the 1000 mg dose for MTD determination (posterior probability of the true DLT rate was <0.25), the 720 mg Q3W dose was defined as the MTD and RP2D, based on an overall safety data review and PK/PD data.

In patients treated QW, five DLTs were reported in four patients during the MTD evaluation period and all were drug-related serious AEs: Grade 2 proteinuria, which led to discontinuation in a patient with ovarian cancer (120 mg cohort); Grade 3 hypertension in a patient with tracheal sarcoma (180 mg cohort); Grade 3 hypertension and Grade 3 proteinuria, which resulted in discontinuation in a patient with colic adenocarcinoma of the colon (240 mg cohort); and Grade 4 respiratory distress (not associated with pulmonary embolism), which resulted in discontinuation in a patient with adenocarcinoma of the rectum (240 mg cohort). Therefore BI 836880 180 mg QW was determined as the MTD and RP2D.

### Safety and tolerability

All 29 patients treated Q3W reported ≥1 AE during the on-treatment period; the median number of AEs per patient was 7 (range 2-36). The most common AEs were hypertension (89.7%), asthenia (51.7%), and nausea (44.8%). The most common Grade ≥3 AE was hypertension (41.4%). Three patients (10.3%) had AEs leading to death (two patients with metastatic pancreatic cancer and one patient with esophageal cancer); all of these patients died due to progressive disease, and none were considered related to treatment. Five patients discontinued the study drug due to an AE; one was a DLT [drug-related Grade 3 pulmonary embolism (1000 mg dose cohort)].

A total of 22 (75.9%) patients had DRAEs; the most common were hypertension (51.7%), anemia, and asthenia (20.7% each; Table 2). Of the patients with hypertension, six received antihypertensive medication. Grade 3/4 DRAEs occurred in 10 patients (34.5%). The most common Grade 3 DRAE was hypertension (8 patients, 27.6%). There was one Grade 4 DRAE (dyspnea; 720 mg cohort). Two patients had DRAEs leading to discontinuation of study drug [Grade 3 myocarditis (720 mg dose cohort) and Grade 3 pulmonary embolism (reported as a DLT in the 1000 mg dose cohort)].

**Table 2:** Most common DRAEs occurring in ≥5% of patients treated with BI 836880 administered either Q3W or QW (treated set)

AE, *n* (%)	Q3W (*n* = 29)					QW (*n* = 24)				
	All Grades	Grade 1	Grade 2	Grade 3	Grade 4	All Grades	Grade 1	Grade 2	Grade 3	Grade 4
Any DRAE	22 (75.9)	5 (17.2)	7 (24.1)	9 (31.0)	1 (3.4)	17 (70.8)	4 (16.7)	7 (29.2)	5 (20.8)	1 (4.2)
Hypertension	15 (51.7)	1 (3.4)	6 (20.7)	8 (27.6)	0 (0)	5 (20.8)	1 (4.2)	2 (8.3)	2 (8.3)	0 (0)
Asthenia	6 (20.7)	3 (10.3)	2 (6.9)	1 (3.4)	0 (0)	9 (37.5)	4 (16.7)	5 (20.8)	0 (0)	0 (0)
Anemia	6 (20.7)	3 (10.3)	3 (10.3)	0 (0)	0 (0)	1 (4.2)	1 (4.2)	0 (0)	0 (0)	0 (0)
Vomiting	5 (17.2)	5 (17.2)	0 (0)	0 (0)	0 (0)	2 (8.3)	2 (8.3)	0 (0)	0 (0)	0 (0)
Infusion-related reaction	5 (17.2)	4 (13.8)	1 (3.4)	0 (0)	0 (0)	0 (0)	0 (0)	0 (0)	0 (0)	0 (0)
Nausea	5 (17.2)	5 (17.2)	0 (0)	0 (0)	0 (0)	1 (4.2)	1 (4.2)	0 (0)	0 (0)	0 (0)
Proteinuria	2 (6.9)	1 (3.4)	1 (3.4)	0 (0)	0 (0)	4 (16.7)	0 (0)	3 (12.5)	1 (4.2)	0 (0)
Diarrhea	2 (6.9)	1 (3.4)	0 (0)	1 (3.4)	0 (0)	3 (12.5)	3 (12.5)	0 (0)	0 (0)	0 (0)
Hypersensitivity	2 (6.9)	1 (3.4)	1 (3.4)	0 (0)	0 (0)	0 (0)	0 (0)	0 (0)	0 (0)	0 (0)
Increased AST	2 (6.9)	2 (6.9)	0 (0)	0 (0)	0 (0)	1 (4.2)	1 (4.2)	0 (0)	0 (0)	0 (0)
Lymphopenia	2 (6.9)	1 (3.4)	1 (3.4)	0 (0)	0 (0)	1 (4.2)	0 (0)	1 (4.2)	0 (0)	0 (0)
Decreased appetite	1 (3.4)	1 (3.4)	0 (0)	0 (0)	0 (0)	4 (16.7)	3 (12.5)	1 (4.2)	0 (0)	0 (0)
Dyspnea	1 (3.4)	0 (0)	0 (0)	0 (0)	1 (3.4)	3 (12.5)	1 (4.2)	1 (4.2)	1 (4.2)	0 (0)
Headache	0 (0)	0 (0)	0 (0)	0 (0)	0 (0)	3 (12.5)	3 (12.5)	0 (0)	0 (0)	0 (0)
Ejection fraction decreased	0 (0)	0 (0)	0 (0)	0 (0)	0 (0)	2 (8.3)	0 (0)	0 (0)	2 (8.3)	0 (0)
Cardiac failure	0 (0)	0 (0)	0 (0)	0 (0)	0 (0)	2 (8.3)	0 (0)	1 (4.2)	1 (4.2)	0 (0)
Peripheral edema	0 (0)	0 (0)	0 (0)	0 (0)	0 (0)	2 (8.3)	0 (0)	1 (4.2)	1 (4.2)	0 (0)
Pleural effusion	0 (0)	0 (0)	0 (0)	0 (0)	0 (0)	2 (8.3)	2 (8.3)	0 (0)	0 (0)	0 (0)
Upper abdominal pain	0 (0)	0 (0)	0 (0)	0 (0)	0 (0)	2 (8.3)	1 (4.2)	1 (4.2)	0 (0)	0 (0)

Grade 4 DRAE in patients treated QW was respiratory distress. There were no Grade 5 DRAEs.

AE, adverse event; AST, aspartate aminotransferase; DRAE, drug-related adverse event; Q3W, once every 3 weeks; QW, once weekly.

All 24 patients treated QW reported ≥1 AE during the on-treatment period; the median number of AEs per patient was 7 (range 1-34). The most common AEs were asthenia (62.5%), decreased appetite (37.5%), and abdominal pain, constipation, and diarrhea (25.0% each). No patients had AEs leading to death.

Overall, 17 (70.8%) patients had DRAEs (Table 2); the most common were asthenia (37.5%) and hypertension (20.8%). Of the patients who experienced hypertension, four received antihypertensive medication. Related Grade 3/4 events occurred in six patients (25%); the most common Grade 3 events were hypertension and decreased ejection fraction [two patients each (8.3%)]. There was one Grade 4 DRAE (respiratory distress which was reported as DLT; 240 mg cohort). Five patients had DRAEs leading to discontinuation of study drug [three patients with DLTs: proteinuria (*n* = 2) and respiratory distress (*n* = 1); one patient with Grade 3 abdominal infection and one patient with Grade 1 hemoptysis].

### Pharmacokinetics and pharmacodynamics

**Figure 1:**
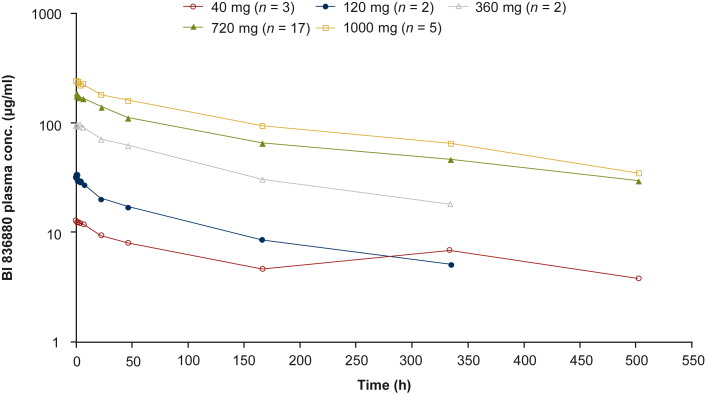
**gMean plasma concentration–time profiles after first infusion (Cycle 1) of BI 836880 administered Q3W.** conc, concentration; gMean, geometric mean; Q3W, once every 3 weeks.

Plasma concentrations of BI 836880 increased with increasing dose in patients treated with both Q3W and QW schedules. Plasma concentration–time profiles are shown in Figure 1 and Supplementary Figure S2, available at https://doi.org/10.1016/j.esmoop.2022.100576. Furthermore, the maximum measured plasma concentration (*C*_max_) and AUC_0–tz_ both increased in a dose-proportional manner over the entire dose range with Q3W and QW treatment (Supplementary Table S1, available at https://doi.org/10.1016/j.esmoop.2022.100576).

**Figure 2:**
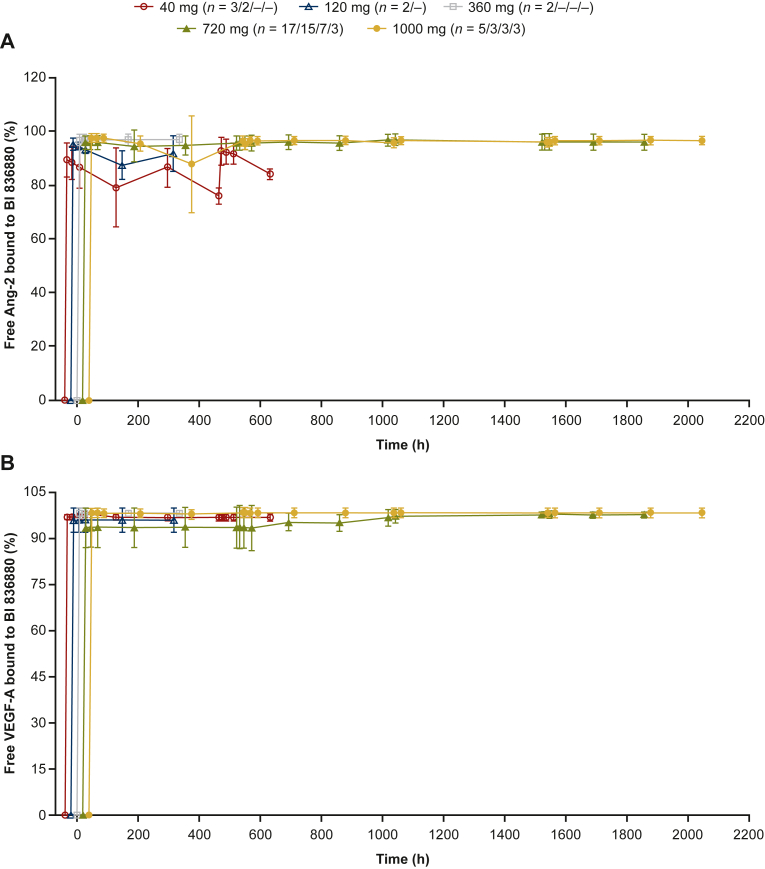
Mean binding-time profiles of (A) Ang-2 and (B) VEGF-A to BI 836880 after multiple Q3W infusions. Ang-2, angiopoietin-2; Q3W, once every 3 weeks; VEGF-A, vascular endothelial growth factor A.

In patients treated Q3W, blood VEGF-A and Ang-2 were almost completely inhibited (>90%) by BI 836880 at the first postdose sampling point (8 hours) of Cycle 1 irrespective of the dose administered for VEGF-A and for doses ≥360 mg Q3W for Ang-2 (Figure 2). VEGF-A and Ang-2 remained completely inhibited over the entire dosing interval and all subsequent cycles. Results from QW dosing were similar (Supplementary Figure S3, available at https://doi.org/10.1016/j.esmoop.2022.100576).

A population PK model for BI 836880 was developed using quantifiable postdose BI 836880 plasma concentrations from patients treated Q3W in Study 1336.1 (Study 1336.6 was ongoing at this time and no data were available to include in the model). A two-compartment PK model provided a good description of BI 836880 plasma concentrations at the population and individual levels. A population PK/PD model was developed to evaluate the effect of BI 836880 on plasma concentrations of Ang-2 for several doses that Boehringer Ingelheim considered to use for the next study. VEGF measurements were not considered for this model, as VEGF-A levels were inhibited >90% for all doses and all samples collected. A mass action model in which BI 836880 reversibly bound Ang-2 and sequestered it in a circulating BI:Ang-2 complex provided a good description of free Ang-2 and total Ang-2 levels. Within this analysis, it was shown that Ang-2 would be completely blocked (>90% inhibition) over the entire dosing interval for doses of 360, 500, and 720 mg Q3W, in 85.7, 95.2, and 100% of patients, respectively. Hence for the large majority of the patients on these two doses (360 and 500 mg) complete Ang-2 binding would be reached. Based on the results, Boehringer Ingelheim decided to include the 500 mg dose into the combination study with the PD-1 inhibitor ezabenlimab (NCT03468426 and NCT03697304).

### Immunogenicity assessment

Of the 24 patients treated with the Q3W schedule who were evaluable for ADAs, 15 (62.5%) showed pre-existing BI 836880 ADAs before the start of treatment. From the nine remaining patients, eight patients (33.3%) developed treatment-induced BI 836880 ADAs and only one patient (4.2%) did not develop ADAs. The time of onset of ADA development was rather fast, usually within 24 days, and only two patients developed BI 836880 ADAs at a later time point.

Of the 23 patient treated with the QW schedule who were evaluable for ADAs, 13 (56.5%) showed pre-existing BI 836880 ADAs before the start of treatment, including 2 patients (8.7%) with ADA-positive samples at baseline only. Of the remaining 10 patients, 8 patients (34.8%) developed treatment-induced BI 836880 ADAs, and 2 patients (8.7%) did not develop ADAs. The onset of ADA development occurred rather quickly, usually within the first or second treatment cycle (21-45 days); only two patients developed BI 836880 ADAs at a later time point (during Cycle 3 or Cycle 4).

In patients treated both Q3W and QW, there was no dose dependency observed in the development of treatment-induced BI 836880 ADAs and the PK of BI 836880 was not influenced by the development of ADAs (Supplementary Figure S4, available at https://doi.org/10.1016/j.esmoop.2022.100576).

### Antitumor activity

**Figure 3:**
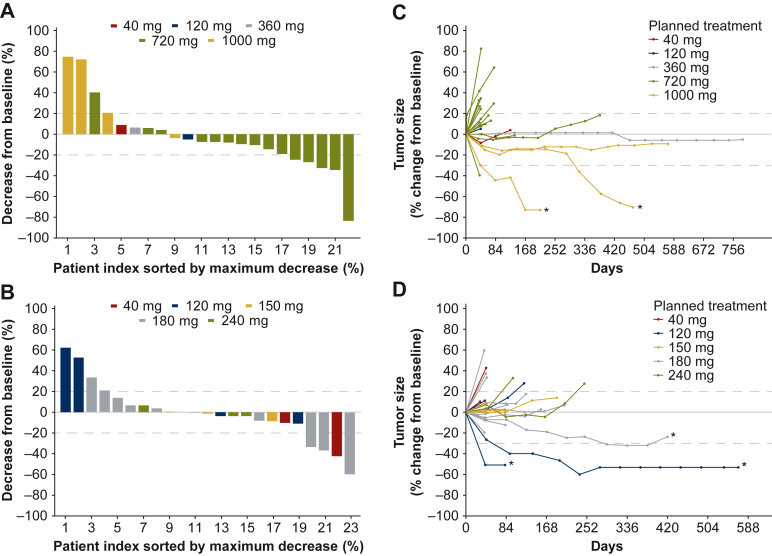
Waterfall plots of best percentage decrease from baseline in sum of target lesion diameter of indicator lesions in patients treated (A) Q3W and (B) QW, and spider plots of target tumor response by dose level in patients treated (C) Q3W and (D) QW. Q3W, once every 3 weeks; QW, once weekly. Positive values represent tumor growth; negative values represent tumor shrinkage. One patient treated Q3W had an unconfirmed partial response (who only had one postbaseline tumor measurement). The asterisk indicates patients with best overall response of confirmed partial response.

A waterfall plot of best percentage decrease from baseline in the sum of the target lesions and a spider plot of tumor response for patients treated with the Q3W and QW treatment schedules are shown in Figure 3.

Of the 22 patients treated Q3W who were evaluable for response, two patients had confirmed partial responses (PRs; both patients received BI 836880 1000 mg). One patient (previously treated with sorafenib) had cavum carcinoma (duration of response: 12.1 weeks) and the other patient (previously treated with bevacizumab) had ovarian adenocarcinoma (duration of response: 5.6 weeks). In addition, one patient with HER2-negative breast cancer in the 720 mg cohort had an unconfirmed PR (the patient had only one postbaseline tumor measurement). Nine patients had stable disease (SD), including three with SD lasting ≥6 months [mucinous adenocarcinoma of unknown origin (360 mg cohort), sigmoid adenocarcinoma (720 mg cohort), and neuroectodermal tumor of the left iliac fossa (1000 mg cohort)]. The patients with mucinous adenocarcinoma and neuroectodermal tumor were on treatment at data cut-off, with no evidence of progressive disease (last tumor measurement on Days 782 and 571, respectively). Progressive disease was the best response for 10 patients; confirmed response was not evaluable due to missing data in 8 patients (including the patient with an unconfirmed PR).

Of 23 patients treated QW who were evaluable for response, 3 patients had confirmed PRs. One patient had endometrial adenocarcinoma (120 mg cohort; duration of response: 84.6 weeks); one had ovarian papillary serous carcinoma (120 mg cohort; duration of response was not available as there were no further imaging data); and one had mucinous colorectal adenocarcinoma (180-mg cohort; duration of response: 12.1 weeks). Nine patients had SD, including three patients with SD lasting ≥6 months [breast invasive ductal carcinoma (150 mg cohort), rectal adenocarcinoma (180 mg cohort), and gastric adenocarcinoma (240 mg cohort)].

## Discussion

Here, we summarize results from two phase I studies of BI 836880, a VEGF/Ang-2 inhibitor, which aimed to define the MTD and RP2D of BI 836880 administered Q3W or QW in patients with advanced or metastatic solid tumors. The MTD was determined as BI 836880 720 mg Q3W and as BI 836880 180 mg QW. There was no evidence of dose-dependent increases in safety risk in patients treated either Q3W or QW, while responses tended to be observed in the higher-dose cohorts. Based on population PK/PD model simulations, most patients were predicted to reach >90% free Ang-2 inhibition over the complete treatment cycle at steady-state at the 500 mg Q3W and 720 mg Q3W dose levels (91.4% and 95.6% of patients, respectively). VEGF-A measurements were not considered in the population PK/PD model, as free VEGF-A levels were inhibited >90% for all doses and all samples collected.

Treatment with BI 836880 was generally well tolerated and there was no dose-dependent increase in the frequency of DRAEs. The most common DRAEs were hypertension and asthenia in patients treated Q3W and QW, and anemia in patients treated Q3W. The AE profile was as expected for an agent targeting VEGF and Ang-2. Hypertension is a well-recognized side-effect of VEGF inhibition,^15^ and has been documented in studies of bevacixumab^16^, ^17^, ^18^, ^19^, ^20^ and vanucizumab.^21^

BI 836880 showed typical PK/PD for a nanobody. Plasma concentrations of BI 836880 increased with increasing dose, and plasma concentration–time profiles were similar among doses. In line with the mechanism of action of BI 836880, VEGF-A and Ang-2 were almost completely bound (>90%) by BI 836880 at the first postdose sampling point of Cycle 1 and remained completely bound over the entire dosing interval and all subsequent cycles. Similarly, in a study investigating the bispecific Ang-2 and VEGF antibody vanucizumab, levels of both biomarkers decreased rapidly following treatment.^21^

The majority of patients developed ADAs to BI 836880 (95.8% and 91.3% of patients treated Q3W and QW, respectively). These ADAs were most likely not directly targeted against the paratopes, and rather were directed against the C terminus of BI 836880, as suggested by the rapid onset of production and the high percentage of patients with pre-existing ADAs. Further characterization of the domain specificity might be required to confirm this interpretation.

Early signs of clinical activity were observed in the two current studies that enrolled a heterogeneous population of patients, all of whom had advanced solid tumors refractory to available therapy. Two patients treated Q3W and three patients treated QW achieved PRs. Of note, both patients who received the Q3W schedule had previously been treated with antiangiogenic therapies (patient with carcinoma of the cavum, sorafenib; patient with ovarian adenocarcinoma, bevacizumab). Furthermore, durable SD was observed in some patients, with two patients treated Q3W remaining on treatment at the time of data cut-off. Symptomatic progression was reported after the cut-off date for the final report: progression-free survival was 1578 days (360 mg) and 1321 days (1000 mg), respectively.

In a previous study investigating vanucizumab in 42 patients with advanced solid tumors, two patients achieved PR, 19 patients had SD, and 10 patients were without disease progression for ≥6 months.^21^ However, in a phase II study in patients with previously untreated metastatic colorectal cancer, vanucizumab plus chemotherapy did not improve progression-free survival versus bevacizumab plus chemotherapy.^22^ Furthermore, vanucizumab was associated with increased rates of antiangiogenic toxicity, such as hypertension and peripheral edema. Development of vanucizumab as a monotherapy was discontinued following this study.^22^ Vanucizumab is a bispecific monoclonal antibody, whereas BI 836880 is a nanobody.

VEGF and Ang-2 also have distinct immunosuppressive effects in the tumor microenvironment. VEGF inhibits dendritic cell maturation and T cell function, and promotes the activity of regulatory T cell and myeloid-derived suppressor cells.^11^^,^^23^ Ang-2 increases the recruitment and adhesion of neutrophils and Tie-2-expressing macrophages (TEMs) to the endothelium, and increases their conversion to the M2-like macrophage phenotype. It also stimulates TEMs to secrete interleukin-10, which promotes regulatory T-cell expansion and the inhibition of effector T cells.^11^ As such, there is interest in combining antiangiogenic agents with PD-1 or programmed death-ligand 1 inhibitors. Combination of anti-VEGF therapy with PD-1 pathway inhibition has been shown to be effective in several tumor types.^24^^,^^25^ As Ang-2 leads to immune suppression by a different mechanism to VEGF,^11^ it is anticipated that addition of Ang-2 inhibition may further improve antitumor activity.

### Conclusion

In the current studies, the MTD of BI 836880, a humanized bispecific nanobody® comprising two single variable domains that inhibit VEGF and Ang-2, was determined as 720 mg Q3W and 180 mg QW. Therefore 720 mg Q3W was chosen as the RP2D for the monotherapy studies. BI 836880 was generally manageable and demonstrated preliminary antitumor activity; the findings from these phase I, single-arm, nonrandomized studies therefore warrant further investigation. Ongoing studies will examine the safety and antitumor activity of BI 836880 and other anticancer agents given in combination with the PD-1 inhibitor ezabenlimab in advanced solid tumors (NCT03468426 and NCT03697304).^26^, ^27^, ^28^, ^29^, ^30^ As ezabenlimab is given in a Q3W schedule, and the QW schedule of BI 836880 provided no advantage over the Q3W schedule in terms of target engagement in the current studies, BI 836880 Q3W will be investigated. Based on overall assessment of efficacy, safety, and PK/PD from the current studies, the following doses were selected for the dose-finding phase of the combination study: 360, 500, and 720 mg Q3W.

## References

[1] Li T., Kang G., Wang T., Huang H. (2018). Tumor angiogenesis and anti-angiogenic gene therapy for cancer. Oncol Lett.

[2] El-Kenawi A., El-Remessy A. (2013). Angiogenesis inhibitors in cancer therapy: mechanistic perspective on classification and treatment rationales. Br J Pharmacol.

[3] Fukumura D., Kloepper J., Amoozgar Z., Duda D.G., Jain R.K. (2018). Enhancing cancer immunotherapy using antiangiogenics: opportunities and challenges. Nat Rev Clin Oncol.

[4] Garcia J., Hurwitz H.I., Sandler A.B. (2020). Bevacizumab (avastin) in cancer treatment: a review of 15 years of clinical experience and future outlook. Cancer Treat Rev.

[5] Syed Y.Y., McKeage K. (2015). Aflibercept: a review in metastatic colorectal cancer. Drugs.

[6] Qin S., Li A., Yi M., Yu S., Zhang M., Wu K. (2019). Recent advances on anti-angiogenesis receptor tyrosine kinase inhibitors in cancer therapy. J Hematol Oncol.

[7] Hashizume H., Falcón B.L., Kuroda T. (2010). Complementary actions of inhibitors of angiopoietin-2 and VEGF on tumor angiogenesis and growth. Cancer Res.

[8] Hu B., Cheng S.Y. (2009). Angiopoietin-2: development of inhibitors for cancer therapy. Curr Oncol Rep.

[9] Hofmann I, Baum A, Hilberg F, et al. Dual targeting of angiogenesis pathways: combined blockade of VEGF and Ang2 signaling. Data presented at the 8th Euro Global Summit on Cancer Therapy. November 3-5, 2015; Valencia, Spain.

[10] Reginato S., Gianni-Barrera R., Banfi A. (2011). Taming of the wild vessel: promoting vessel stabilization for safe therapeutic angiogenesis. Biochem Soc Trans.

[11] Rahma O.E., Hodi F.S. (2019). The intersection between tumor angiogenesis and immune suppression. Cancer Res.

[12] Tsutsui S., Inoue H., Yasuda K. (2006). Angiopoietin 2 expression in invasive ductal carcinoma of the breast: its relationship to the VEGF expression and microvessel density. Breast Cancer Res Treat.

[13] Andersen S., Donnem T., Al-Shibli K. (2011). Prognostic impacts of angiopoietins in NSCLC tumor cells and stroma: VEGF-A impact is strongly associated with Ang-2. PLoS One.

[14] Brown J.L., Cao Z.A., Pinzon-Ortiz M. (2010). A human monoclonal anti-ANG2 antibody leads to broad antitumor activity in combination with VEGF inhibitors and chemotherapy agents in preclinical models. Mol Cancer Ther.

[15] Hayman S.R., Leung N., Grande J.P., Garovic V.D. (2012). VEGF inhibition, hypertension, and renal toxicity. Curr Oncol Rep.

[16] Ogita S., Tejwani S., Heilbrun L. (2012). Pilot Phase II trial of bevacizumab monotherapy in nonmetastatic castrate-resistant prostate cancer. ISRN Oncol.

[17] Kreisl T.N., Zhang W., Odia Y. (2011). A phase II trial of single-agent bevacizumab in patients with recurrent anaplastic glioma. Neuro Oncol.

[18] Cannistra S.A., Matulonis U.A., Penson R.T. (2007). Phase II study of bevacizumab in patients with platinum-resistant ovarian cancer or peritoneal serous cancer. J Clin Oncol.

[19] Burger R.A., Sill M.W., Monk B.J., Greer B.E., Sorosky J.I. (2007). Phase II trial of bevacizumab in persistent or recurrent epithelial ovarian cancer or primary peritoneal cancer: a Gynecologic Oncology Group Study. J Clin Oncol.

[20] Cobleigh M.A., Langmuir V.K., Sledge G.W. (2003). A phase I/II dose-escalation trial of bevacizumab in previously treated metastatic breast cancer. Semin Oncol.

[21] Hidalgo M., Martinez-Garcia M., Le Tourneau C. (2018). First-in-human phase I study of single-agent vanucizumab, a first-in-class bispecific anti-angiopoietin-2/anti-VEGF-A antibody, in adult patients with advanced solid tumors. Clin Cancer Res.

[22] Bendell J.C., Sauri T., Gracián A.C. (2020). The McCAVE trial: vanucizumab plus mFOLFOX-6 versus bevacizumab plus mFOLFOX-6 in patients with previously untreated metastatic colorectal carcinoma (mCRC). Oncologist..

[23] Yang J., Yan J., Liu B. (2018). Targeting VEGF/VEGFR to modulate antitumor immunity. Front Immunol.

[24] Finn R., Qin S., Ikeda M. (2020). Atezolizumab plus bevacizumab in unresectable hepatocellular carcinoma. N Engl J Med.

[25] Socinski M.A., Jotte R.M., Cappuzzo F. (2018). Atezolizumab for first-line treatment of metastatic nonsquamous NSCLC. N Engl J Med.

[26] Girard N., Wermke M., Barlesi F. (2021). Phase Ib study of BI 836880 (VEGF/Ang2 nanobody) plus ezabenlimab (BI 754091; anti-PD-1 antibody) in patients (pts) with solid tumors. J Clin Oncol.

[27] Girard N., Wermke M., Barlesi F. (2021). PD1-1-1 Phase Ib study of BI 836880 (VEGF/Ang2 nanobody®) plus ezabenlimab (BI 754091, anti-PD-1 antibody) in patients with solid tumors. Ann Oncol.

[28] Hussein M.A., Percent I.J., Bendell J.C. (2021). Platform trial of ezabenlimab (BI 754091), an anti-PD-1 antibody, in patients (pts) with previously treated advanced solid tumors: combination with BI 836880, a VEGF/Ang2-blocking nanobody. J Clin Oncol.

[29] Hussein M.A., Bendell J.C., Arkenau H.T. (2021). Platform trial of BI 754091, an anti-PD-1 antibody, in patients with previously treated advanced solid tumors: combination with BI 836880, a VEGF/Ang2-blocking nanobody. J Clin Oncol.

[30] Patel M., Johnson M., Winer I. (2021). 542P Ezabenlimab (BI 754091) monotherapy in patients (pts) with advanced solid tumours. Ann Oncol.

