# Supplementary material for: Two phase I studies of BI 836880, a vascular endothelial growth factor/angiopoietin-2 inhibitor, administered once every 3 weeks or once weekly in patients with advanced solid tumors
Source: ESMO Open. 2022 Sep 13;7(5):100576. doi: 10.1016/j.esmoop.2022.100576 (PMC9588896; doi:10.1016/j.esmoop.2022.100576)
Supplement: Supplementary Material [file mmc1.docx]

**Supplementary Materials**

The following adverse events (AEs) qualified as dose-limiting toxicities (DLTs):

- Drug-related Common Terminology Criteria for Adverse Events (CTCAE) Grade ≥3 non-hematological toxicity except:
- Vomiting or diarrhea responding to supporting treatment;
- Fatigue lasting for less than four days;
- Transient Grade 3 infusion reaction (i.e., if infusion-related reaction could be controlled by appropriate medication according to the investigator’s decision and the next infusion was not delayed for more than two weeks);
- Any laboratory abnormality, which was considered not clinically relevant by the investigator or resolved spontaneously or could be resolved with appropriate treatment.
- CTCAE Grade 4 neutropenia more than seven days or complicated by infection.
- CTCAE Grade ≥3 febrile neutropenia.
- CTCAE Grade 4 thrombocytopenia.
- CTCAE Grade ≥3 thrombocytopenia with bleeding.
- CTCAE Grade ≥3 proteinuria (urinary protein ≥3.5 g/day).
- Hypertension: increase of diastolic blood pressure by 15 mmHg confirmed by a second measurement or ambulatory blood pressure measurement (when indicated; e.g., white coat effect) which could not be controlled by anti-hypertensive medication and required a dose reduction of BI 836880 for a further treatment cycle.
- All related AEs that led to an interruption of BI 836880 for more than 14 days until recovery to baseline.
