# Supplementary material for: Two phase I studies of BI 836880, a vascular endothelial growth factor/angiopoietin-2 inhibitor, administered once every 3 weeks or once weekly in patients with advanced solid tumors
Source: ESMO Open. 2022 Sep 13;7(5):100576. doi: 10.1016/j.esmoop.2022.100576 (PMC9588896; doi:10.1016/j.esmoop.2022.100576)

**Supplementary Figure S1.** Patient disposition.

Abbreviations: AE, adverse event; DLT, dose-limiting toxicity; QW, once weekly; Q3W, once every 3 weeks.


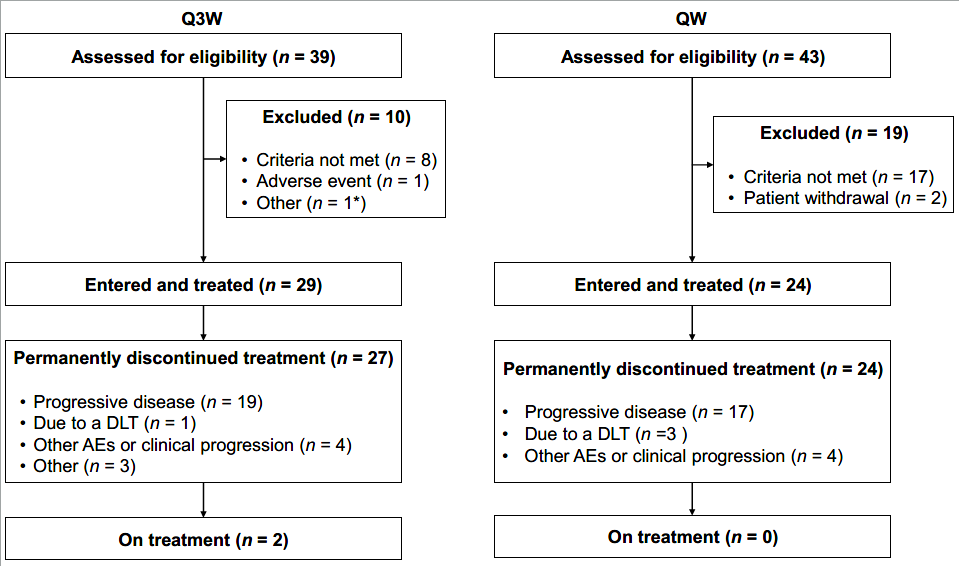


*Other: due to cardiac insufficiency New York Heart Association Grade II and left ventricular ejection fraction of 40%.

**Supplementary Figure S2.** gMean plasma concentration–time profiles of BI 836880 after multiple QW infusions.

Abbreviations: conc, concentration; gMean, geometric mean; QW, once weekly.


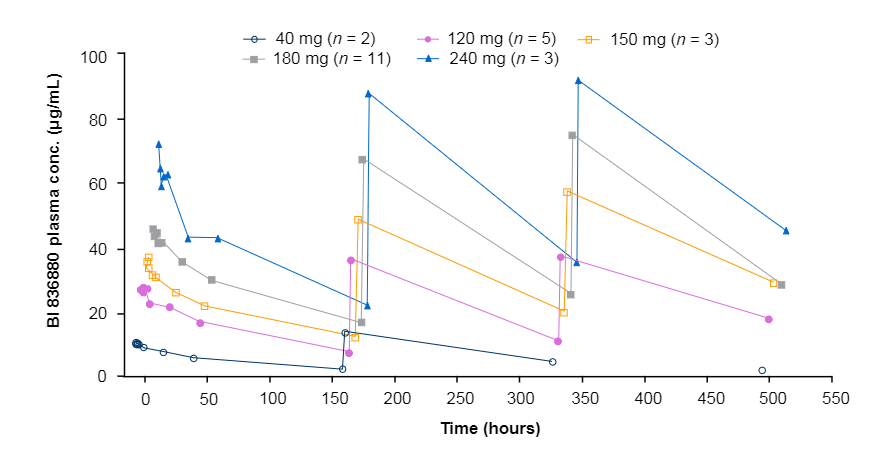


**Supplementary Figure S3.** Mean binding-time profiles of free Ang-2 (**A**) and free VEGF-A (**B**) to BI 836880 after multiple QW infusions.

Abbreviations: Ang-2, angiopoietin-2; QW, once weekly; VEGF-A, vascular endothelial growth factor A.


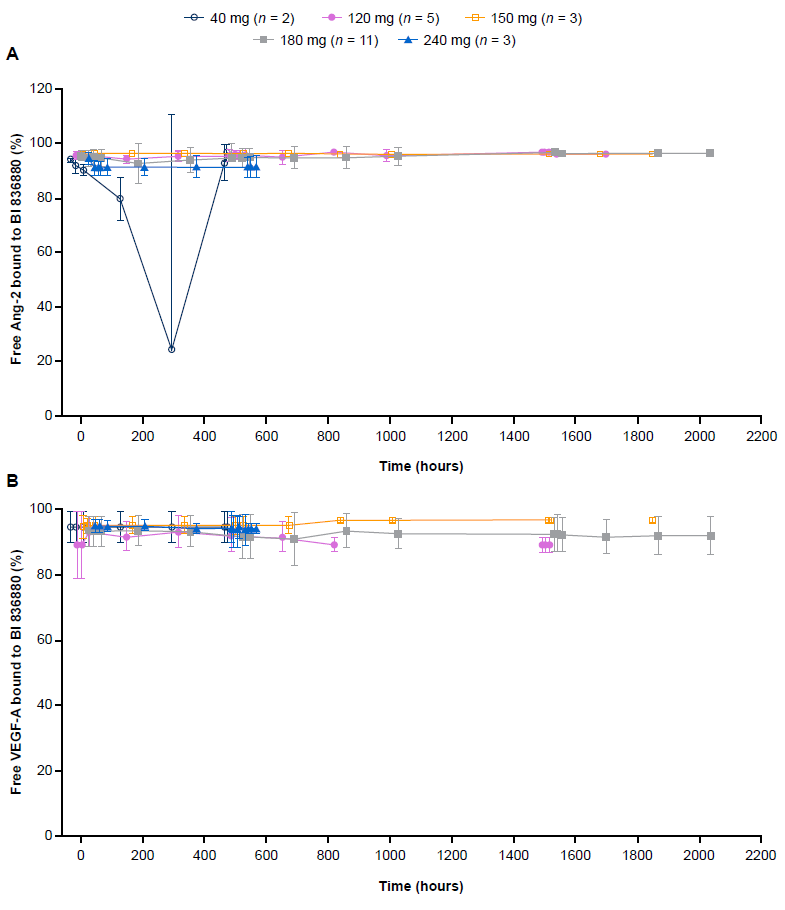


**Supplementary Figure S4.** Comparison of individual AUC_0-tz,norm_ or AUC_0-168h,norm_ values of BI 836880 by overall anti-drug antibody status in Cycle 1 in patients treated A) Q3W and
B) QW.

Abbreviations: ADAs, anti-drug antibodies; AUC, area under the curve; QW, once weekly; Q3W, once every 3 weeks.


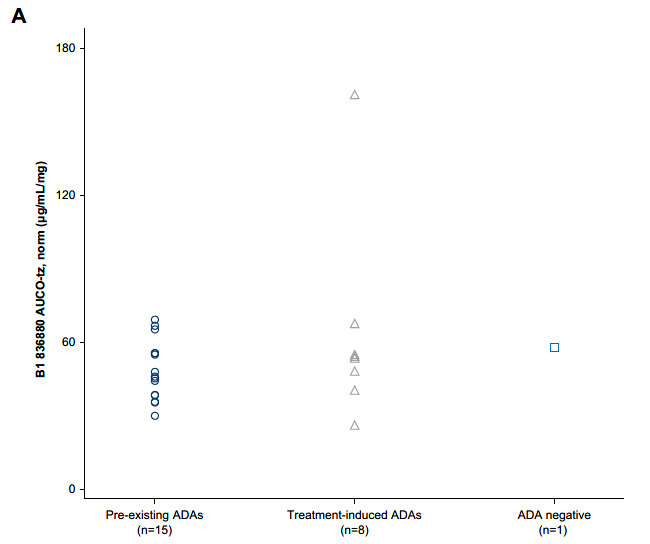


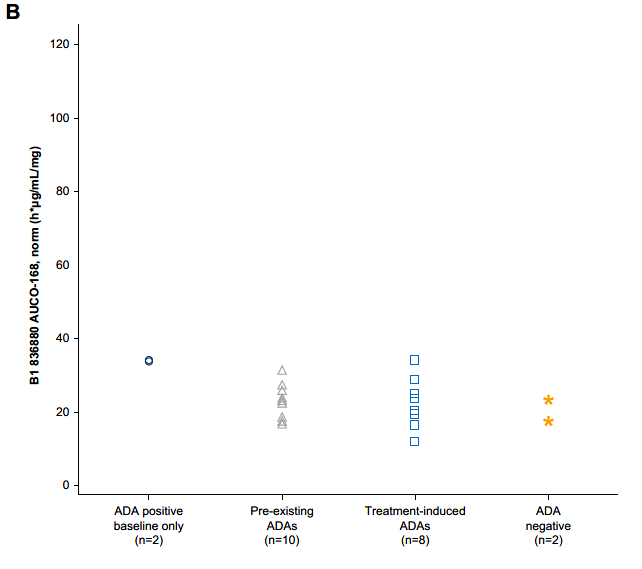

Supplement: Supplementary Figures [file mmc2.docx]
