# Supplementary material for: Two phase I studies of BI 836880, a vascular endothelial growth factor/angiopoietin-2 inhibitor, administered once every 3 weeks or once weekly in patients with advanced solid tumors
Source: ESMO Open. 2022 Sep 13;7(5):100576. doi: 10.1016/j.esmoop.2022.100576 (PMC9588896; doi:10.1016/j.esmoop.2022.100576)
Supplement: Supplementary Tables [file mmc3.docx]

**Supplementary Table 1.** Pharmacokinetic parameters of BI 836880 after Cycle 1 in patients treated Q3W and QW (PK analysis set).

|  | **Q3W** | | | | | **QW** | | | | |
| --- | --- | --- | --- | --- | --- | --- | --- | --- | --- | --- |
|  | **40 mg**  ***n* = 3** | **120 mg**  ***n* = 2** | **360 mg**  ***n* = 2** | **720 mg**  ***n* = 17** | **1000 mg**  ***n* = 5** | **40 mg**  ***n* = 2** | **120 mg**  ***n* = 5** | **150 mg**  ***n* = 3** | **180 mg**  ***n* = 11** | **240 mg**  ***n* = 3** |
| gMean C_max_, μg/mL (gCV%) | 13.7  (29.3) | 34.9 (1.2) | 107 (20.4) | 204 (18.9) | 259 (39.4) | 11.4 (21.6) | 28.6 (35.4) | 38.7 (8.0) | 49.5 (27.3) | 70.3 (40.2) |
| gMean AUC_0-tz,_ μg∙h/mL (gCV%) | 2180 (152.0) | 4100 (38.7) | 15000 (71.1) | 32900 (28.1) | 40000 (66.6) | 950 (10.2) | 1720 (62.2) | 3280 (9.7) | 4190 (46.9) | 5910 (29.5) |

Abbreviations: AUC_0-tz_, area under the plasma concentration–time curve over the time interval from 0 up to the last quantifiable data point; C_max_, maximum measured plasma concentration; gCV, geometric coefficient of variation; gMean, geometric mean; PK, pharmacokinetic; QW, once weekly; Q3W, once every 3 weeks.
